# Genetic Homogeneity of *Francisella tularensis* subsp. *mediasiatica* Strains in Kazakhstan

**DOI:** 10.3390/pathogens13070581

**Published:** 2024-07-12

**Authors:** Alexandr Shevtsov, Uinkul Izbanova, Asylulan Amirgazin, Alma Kairzhanova, Ayan Dauletov, Vladimir Kiyan, Gilles Vergnaud

**Affiliations:** 1 National Center for Biotechnology, Astana 010000, Kazakhstan; amirgazin@biocenter.kz (A.A.); kairzhanova@biocenter.kz (A.K.); dauletov@biocenter.kz (A.D.); vskiyan@gmail.com (V.K.); 2 Aikimbayev’s National Scientific Center for Especially Dangerous Infections, Almaty 050000, Kazakhstan; 3 Institute for Integrative Biology of the Cell (I2BC), Université Paris-Saclay, CEA, CNRS, 91198 Gif-sur-Yvette, France

**Keywords:** *Francisella tularensis* subsp. *mediasiatica*, whole genome sequencing, genotyping, MLVA, Kazakhstan

## Abstract

Tularemia is an acute febrile disease caused by the Gram-negative bacillus *Francisella tularensis*. Based on genetic and phenotypic characteristics, three subspecies are distinguished: *tularensis*, *holarctica*, and *mediasiatica*. *F. tularensis* subsp. *mediasiatica* remains the least studied subspecies. Over the past decade, new foci of distribution of *F. tularensis* subsp. *mediasiatica* have been discovered in Russia (Siberia), expanding the possible distribution area by thousands of kilometers. This article provides whole genome single nucleotide polymorphism (wgSNP) and polymorphic tandem repeats (MLVA) analyses of 28 *mediasiatica* strains isolated between 1965 and 2004 in Kazakhstan. Despite high genetic homogeneity, MLVA with eleven loci (MLVA11) demonstrates a high discriminatory ability (diversity index, 0.9497). The topological structure of the trees based on wgSNP and MLVA is not comparable; however, clustering remains congruent for most outbreaks, with the exception of two strains from one outbreak that are identical in terms of wgSNP but differ at three tandem repeat loci. Based on wgSNP, the strains are assigned to one of the three currently known *mediasiatica* sublineages, lineage M.I, together with other historical strains maintained in collections in Russia and Sweden. wgSNP shows limited previously unknown genetic diversity, with the M.I lineage size being only 118 SNPs. The wgSNP genotype is not strongly correlated with year and place of isolation.

## 1. Introduction

Tularemia is an acute febrile disease, the form and severity of which depend on the route of infection, subtype, and subpopulation of the pathogen [1,2]. The causative agent of tularemia is *Francisella tularensis*, which includes three subspecies, *tularensis*, *holarctica*, and *mediasiatica*, differing in geographical distribution and pathogenicity. *F. tularensis* subsp. *tularensis*, present in North America, is the most pathogenic for humans with an infectious dose lower than 10 colony forming units (CFU) and potential for use as a biological weapon [3,4,5]. *F. tularensis* subsp. *holarctica*, considered endemic to the northern hemisphere, is present in North America and across Eurasia [6,7]. *F. tularensis* subsp. *holarctica* biovar *japonica* was detected in Australia [8]. Suspicion of the presence of *F. tularensis* in North Africa (Algeria) was recently published [9]. *F. tularensis* subsp. *mediasiatica* was originally described by Aikimbayev and discovered in the Central Asian region of the USSR (Kazakhstan, Uzbekistan) [10,11]. The genetic diversity of subspecies *tularensis* and *holarctica* is well documented in the literature due to their wider distribution and established pathogenicity to humans [12,13,14,15,16,17]. The limited distribution of subspecies *mediasiatica* to the Central Asian region and the lack of documented cases of human infection have led to a limited number of publications on its genetic diversity.

The identification of new foci of distribution of subspecies *mediasiatica* in the Altai and Krasnoyarsk territories of Russia indicates a broader geographical range [18,19]. Experiments conducted on laboratory animals have demonstrated an intermediate level of virulence of *F. tularensis* subsp. *mediasiatica* between the species *tularensis* and *holarctica*, contributing to an increased interest in studying this subspecies [20]. Currently, full-genome genotyping has been performed on strains circulating in Russia and those isolated in Central Asia, which are deposited in microorganism collections in Russia and/or Sweden [21,22].

Although *F. tularensis* subsp. *mediasiatica* has been known in Central Asia since the 1960s, there is limited information about its genetic diversity in the region. The goal of this work was to conduct full-genome sequencing of strains collected and deposited in Kazakhstan.

## 2. Materials and Methods

### 2.1. Strain and DNA Extraction

This work used strains of *F. tularensis* subsp. *mediasiatica* deposited in the Masgut Aikimbayev National Scientific Center for Especially Dangerous Infections (NSCEDI) (Almaty, Kazakhstan) under the Ministry of Healthcare of the Republic of Kazakhstan. After initial isolation and identification, the strains were stored in lyophilized form. For the study, lyophilized strains were reconstituted in sterile distilled water and cultured on selective “FT-agar” (FBIS SRCAMB, Obolensk, Russia) [23] at 37 °C for 48 h. The bacterial mass was harvested and inactivated following established procedures [16]. DNA extraction from the inactivated culture was performed using the QIAamp DNA Mini Kit (Qiagen, Hilden, Germany), following the manufacturer’s instructions.

### 2.2. Multiple Loci VNTR (Variable Number of Tandem Repeats) Analysis (MLVA) Typing

MLVA genotyping was conducted using eleven VNTR loci, Ft-M02, Ft-M03, Ft-M04, Ft-M05, Ft-M06, Ft-M10, Ft-M20A, Ft-M20B, Ft-M22, Ft-M23, and Ft-M24. The distribution of reaction mixtures, primer concentrations, and amplification mode followed the procedures outlined in the original MLVA genotyping report [24]. The composition of the reaction mixture varied based on the reagents available for the study and included the following: primers, 20 mM (NH_4_)_2_SO_4_, 0.01% Tween 20, 2.5 mM MgCl_2_, 200 nM of each dNTP, 5% DMSO, Taq DNA Polymerase (Thermo Scientific™, Vilnius, Lithuania), and Pfu DNA Polymerase (Promega, Madison, WI, USA) in a ratio of 1.9 U:0.1 U, along with 10 ng of template DNA.

PCR products were separated on a DNA Analyzer 3730xl (Applied Biosystems, Hitachi, Japan, Tokyo), employing GeneScan™ 1200 LIZ (Applied Biosystems, Vilnius, Lithuania) as size standard. Visualization and allele assignment were conducted using the GeneMapper 4.0 software. The Pearson correlation coefficient and UPGMA clustering method were applied using BioNumerics 8.1 (Applied Maths, Sint-Martens-Latem, Belgium) to visualize clustering relations. Simpson’s index was utilized to assess the discrimination of VNTR loci and MLVA11 [25].

### 2.3. Genome Sequencing, Whole Genome Single Nucleotide Polymorphism (wgSNP) Analysis

DNA concentration was determined using the Qubit dsDNA HS Assay Kit and Qubit dsDNA BR Assay Kit (Invitrogen™, Carlsbad, CA, USA) with a Qubit 2.0 Fluorometer (Life Technologies, Tecan, Grödig, Austria). DNA library preparation was conducted using the Illumina DNA Prep (M) Tagmentation kit (Catalog No. 20060060, Illumina, San Diego, CA, USA), following the manufacturer’s instructions. Sequencing was performed on the MiSeq sequencer platform (Illumina, San Diego, CA, USA) using the MiSeq Reagent Kit v3, 600 Cycles (Catalog No. MS-102-3003).

The resulting reads were evaluated for contamination using the discoSnp++ version 2.3 software [26]. Raw data were then assembled into contigs using SKESA [27]. Assembly metrics and sequencing results were evaluated using the n50 software within the SeqFu package v1.20.3 [28]. The assembled contigs were employed to construct a phylogeny based on single nucleotide polymorphisms (SNPs) using BioNumerics software version 8.1 (Applied Maths, Sint-Martens-Latem, Belgium), following established protocols [29]. The contigs were split in 50 bp long in silico reads, which were mapped on a reference genome (GCA_000018925 corresponding to *F. tularensis* subsp. *mediasiatica* FSC147) using BioNumerics default parameters. SNPs were subsequently called using the “Strict SNP filtering (Closed SNP set)” SNP analysis template. This template recovers core-genome SNPs with a minimum inter-SNP distance of 12 bp.

## 3. Results

### 3.1. Characteristics of Strains

**Figure 1:**
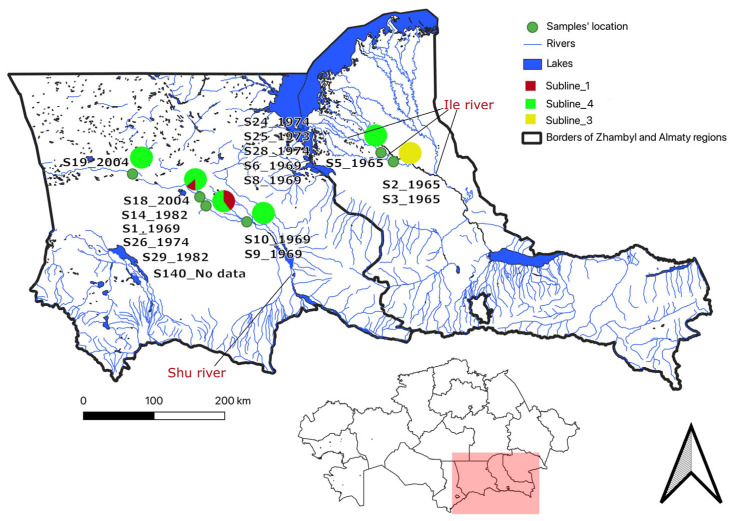
Precise sample collection location data from 17 strains. Three *mediasiatica* strains were collected in 1965 in two locations (Kokushbay and Jeltorangy) separated by 29 km within the Ili river floodplain, Almaty region. Fourteen were collected between 1969 and 2004 in four locations (Kumozek, Karaboget, Ulanbel, Moiynkum) distributed over 170 km within the Shu river floodplain, Jambyl (Zhambyl) region.

DNA was isolated from 28 *F. tularensis mediasiatica* strains deposited in the NSCEDI collection. The strains were collected between 1965 and 2004 from two southern regions of Kazakhstan (Jambyl and Almaty) as part of regular monitoring studies (Table S1). Strains were recovered in eight years during this forty-year period, with a maximum of six strains collected in years 1969 and 1982. The exact geographical locations are known for 17 strains. They correspond to four and two collection points in the floodplains of the Shu (Jambyl region) and Ili (Almaty region) rivers, respectively (Figure 1). Eighteen strains were isolated from tick suspensions and four from suspensions of rodent organs (*Lepus tolai* (Tolai hare), *Meriones unguiculatus* (Mongolian gerbil), *Mus musculus* (house mouse), *Rhombomys opimus* (great gerbil)) caught in tularemia foci. No information was available regarding the year of isolation or host for one and six strains, respectively (Table S1).

### 3.2. MLVA Genotyping

The eleven VNTR loci were successfully amplified in the 28 *F. tularensis* subsp. *mediasiatica* strains (Table S1). Significantly high variability was observed in the Ft-M03, Ft-M05, and Ft-M22 loci, with 14, 7, and 7 alleles identified, respectively. The Simpson diversity index for these loci was 0.9153, 0.7963, and 0.8016, respectively (Table 1). Two alleles were observed at the Ft-M06 and Ft-M20A loci, yielding diversity indices of 0.1376 and 0.0714, respectively. The remaining six loci showed no variation.

**Figure 2:**
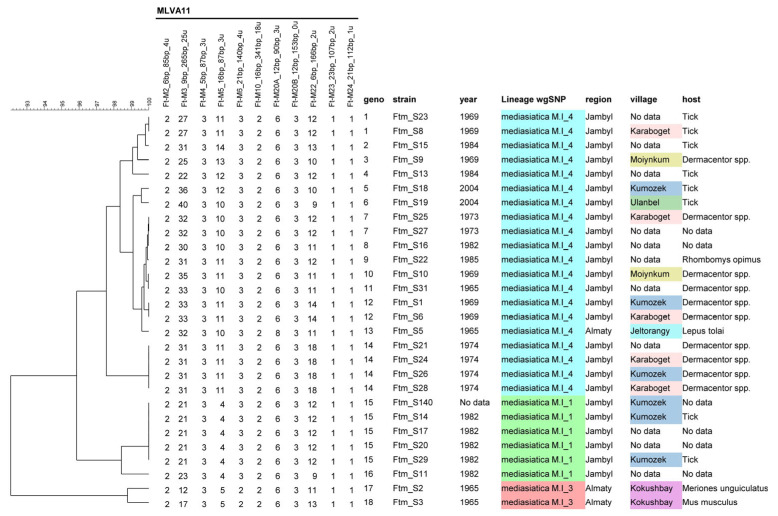
Multiple-locus VNTR analysis (MLVA11) clustering of the 28 strains *F. tularensis* subsp. *mediasiatica* isolated in Kazakhstan. The 18 genotypes are numbered in the “geno” column. The lineage assignment deduced from whole-genome SNP analysis is included and colored to illustrate the global congruence of the two approaches. A distinct color is used for each sampling site in the “village” column.

The eleven VNTR loci resolved 18 genotypes, yielding a diversity index of 0.9497 (Table 1, Figure 2). Thirteen genotypes were unique, while three genotypes each comprised two strains, and two genotypes combined four or five strains. Notably, genotypes comprising two or more strains with available isolation coordinates typically consisted of strains obtained in the same year, from the same site, or located within a short distance (15 km separate Karaboget from Kumozek).

### 3.3. Short-Read Whole-Genome Sequencing and Phylogenetic Analysis

We conducted short-read sequencing of 28 *F. tularensis* subsp. *mediasiatica* strains. On average, each strain yielded 1,155,049 reads (ranging from 478,014 to 1,520,148), resulting in an estimated coverage ranging from 72 to 235 folds (accession numbers are listed in Table S1). Contamination was not detected using the discoSnp++ software. The average size of the assembled genomes was 1,791,980 nucleotides. The number of assembled contigs per strain varied from 74 to 78, with an average N50 value of 34,835 bp (Table S1).

**Figure 3:**
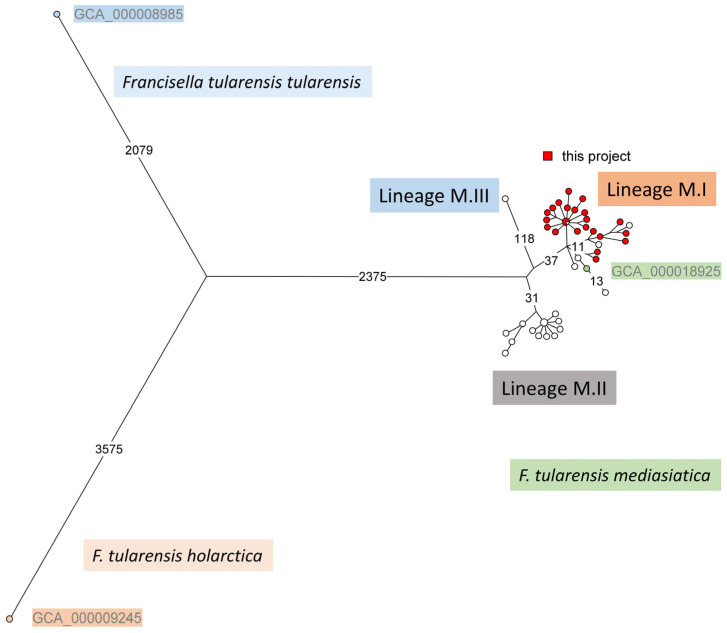
*F. tularensis* subsp. *mediasiatica* wgSNP analysis. Fifty-five WGS datasets, including fifty-three *F. tularensis* subsp. *mediasiatica* and one representative from each of the two other subspecies, *F. tularensis* subsp. *tularensis* and subsp. *holarctica*, were used. A total of 8326 SNPs were called. The maximum parsimony tree had a size of 8341 (homoplasy 0.18%). Branch lengths of more than ten SNPs are indicated. Square root length scaling was used. The *mediasiatica* strain (genome accession GCA_000018925 strain FSC147 lineage M.I) used as a mapping reference for SNP calling is labeled in green. All 28 strains from the present report (labeled in red) are assigned to *mediasiatica* lineage M.I.

A maximum parsimony tree was constructed from the SNP matrix deduced by mapping on genome accession GCA_000018925 (*F. tularensis* subsp. *mediasiatica* strain FSC147). *F. tularensis* subsp. *tularensis* SCHU S4 (GCA_000008985) and *F. tularensis* subsp. *holarctica* LVS (GCA_000009245) were included as outgroups, along with the 28 WGS datasets of this study and 25 additional publicly available *F. tularensis* subsp. *mediasiatica* WGS datasets (Table S2). The tree shows the three *F. tularensis* subsp. *mediasiatica* lineages, M.I, M.II, and M.III, with all strains from this study clustering within lineage M.I (Figure 3).

A total of 8326 SNPs were called, resulting in a tree size of 8341, suggesting that 15 SNPs occur in two branches of the tree, corresponding to a homoplasy level of 0.18%. Given that the nonredundant core genome size of *Francisella* spp. is approximately 1.6 Mb, the expected occurrence of independent mutations at the same position is 17 SNPs [30]. This suggests that most, if not all, homoplastic events reflect random, coincidental mutations. Accordingly, when the two outgroup strains are not included in the analysis, 330 SNPs are called among the 53 *F. tularensis* subsp. *mediasiatica* genomes. The resulting maximum parsimony tree has a size of 330, i.e., without any homoplasy (Figure S1).

The M.I lineage comprised 28 strains from this study and seven previously sequenced strains: strain 240 from the Kazakhstan collection, which was isolated within Kazakhstan; two strains (117 and 120) deposited in Russia but isolated within Kazakhstan; and four strains from the Swedish collection of microorganisms (FSC122, FSC147, FSC148, and FSC149), with one isolated within Kazakhstan and three without a published geographic origin (Table S2). The M.II line consisted of 17 strains isolated in the Krasnoyarsk Territory and the Altai Republic of Russia. The M.III lineage was represented by a single strain isolated in Uzbekistan.

**Figure 4:**
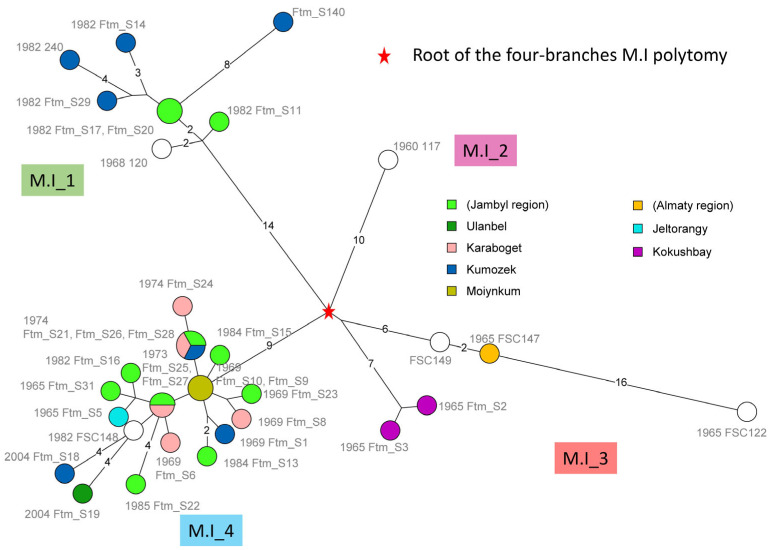
Subspecies *mediasiatica* lineage M.I wgSNP analysis. WGS data from 35 M.I strains were used, including the 28 strains from this project and seven previously published datasets. The maximum parsimony tree is based on 118 SNPs, and the tree size is 119. Branch lengths above one SNP are indicated. The red star indicates the position of the root. The four branches of the polytomy are labeled M.I_1 to M.I_4. Nodes are labeled with strain id (FSC, Swedish collection; Ftm, this report; no prefix, Timofeev 2022 [19]) and colored according to geographic origin when known as indicated. Village names are listed according to their relative position, from the most western (Ulanbel, along the Shu river, Jambyl region) to the most eastern (Kokushbay, along the Ili river, Almaty region), separated by approximately 400 km (Figure 1).

**Figure 5:**
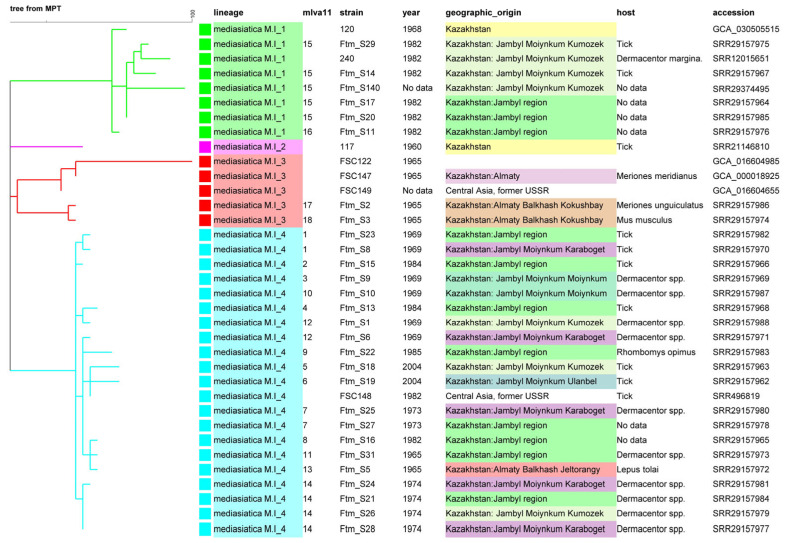
Phylogram view of the subspecies *mediasiatica* lineage M.I wgSNP analysis. The maximum parsimony tree from Figure 4 was rooted. The four branches of the polytomy are colored. A distinct color is used for each sampling site in the “geographic_origin” column. Strain Id, year of isolation, geographic origin, host and WGS data accession numbers are indicated together with the MLVA11 genotype.

The Maximum Parsimony Tree (MPT) constructed from the SNP matrix of these 35 strains of *F. tularensis* subsp. *mediasiatica* lineage M.I has a size of 118 SNPs and is presented in Figure 4 and Figure 5.

The MPTs indicate that while the present collection increases the number of available strains in the M.I lineage fivefold, it reveals limited previously unknown genetic diversity. The M.I lineage is a polytomy with four branches. The M.I_1 branch comprises eight strains, with seven of them isolated in the Jambyl (Zhambyl) region (no geographical data available for strain 120 from the Russian collection). Six strains were isolated in 1982. The largest distance among M.I_1 strains is 14 SNPs. The M.I_2 branch is represented by a single strain (117) deposited in Russia. The M.I_3 branch is further divided into two branches radiating only one SNP from the root: one branch comprises three strains from the Swedish collection, while the other branch is represented by two strains from this study isolated in the Almaty region. Within this sublineage, the maximum genetic distance between strains is 32 SNPs.

The M.I_4 branch within the M.I polytomy is the most populated, comprising 21 strains isolated between 1965 and 2004 from the territories of Jambyl (Zhambyl) and Almaty regions, along with one strain from the Swedish collection lacking an exact geographical reference (FSC148). This sublineage demonstrates the highest genetic homogeneity, with the maximum distance between strains being nine SNPs (Figure 4). Three strains, S31, S5, and S16, isolated in 1965 and 1982, respectively, are separated by only two SNPs. They display a derived genotype compared to two strains (S25, S27) isolated in 1973 and two strains isolated in 1969 (S9, S10). This implies that the 1969 and 1973 genotypes already existed in 1965. Strains S9 and S10 are coincident in terms of the wgSNP genotype. This genotype is ancestral to all the M.I_4 genotypes. It is curious that these two strains differ at three VNTR loci, Ft-M3, Ft-M5, and Ft-M22 (Figure 2 and Table S1).

Genetic diversity can be observed in geographically localized foci. For example, the five strains (S6, S8, S24, S28, and S25) from Karaboget isolated between 1969 to 1974 are relatively dispersed within M.I_4. The six strains from Kumozek were isolated between 1969 and 2004. These strains are part of the M.I_1 or M.I_4 sublineages and are separated by up to 37 SNPs (Figure 1, Figure 4 and Figure 5). The three strains from the Almaty region, isolated in 1965 from two foci located 15 km apart (Jeltorangy and Kokushbay), belong to two different sublineages M.I_3 and M.I_4 and differ by 21 SNPs (Figure 4).

The VNTR clustering appears to be able to correctly predict the assignment to each of the four branches of the M.I polytomy but not at a more precise sub-level (Figure 2 and Figure 5). The clustering based on VNTR is not fully congruent with the wgSNP phylogeny, reflecting a high homoplasy level at the most highly variable locus Ft-M03. Some genetically similar strains on the wgSNP tree and MLVA tree are grouped into the same genotype or clusters. For example, M.I_4 strains Ftm_S21, Ftm_S26, Ftm_S28, Ftm_S25, and Ftm_S27 represent the same genotypes according to both SNP and VNTR analyses, respectively. This is in contrast with strains Ftm_S9 and Ftm_S10, sharing an identical wgSNP genotype but differing at three VNTR loci, despite being isolated in the same year from the same sampling site.

## 4. Discussion

In this study, wgSNP and MLVA11 were employed to genotype 28 strains of *F. tularensis mediasiatica* isolated in Kazakhstan from 1965 to 2004. All strains in this study were isolated from ticks or rodents collected or captured in the floodplains of the Shu and Ili rivers, located in the Almaty and Jambyl (Zhambyl) regions. These natural foci of tularemia, classified by landscape, are referred to as “tugai”. These areas, situated in desert river valleys, are characterized by non-flooded regions with thickets of low-growing trees and shrubs, hence the term “tugai”. More than 80% of the strains in this study were isolated from ticks. In Kazakhstan, the prevalence of ticks infected with *F. tularensis* is 0.3–0.5% [31]. Moreover, ticks constitute over 85% of the samples tested for tularemia, owing to the challenges associated with capturing rodents and ethical considerations.

Historically, this type of focus on the territory of Kazakhstan (along the Shu and Ili rivers) and the territory of Uzbekistan (along the Amur Darya river) was considered to be the sole natural reservoir of the *mediasiatica* subspecies [32,33]. Recently, the *mediasiatica* subspecies has also been detected in the Altai and Krasnoyarsk territories of Russia [21,34], specifically in the “piedmont-stream” foci of tularemia [35,36] located 1500 km away from the foci in Kazakhstan and Uzbekistan. Both types of foci share the presence of freshwater sources. An area of 500 thousand square kilometers in Kazakhstan is endemic for tularemia, with foci of *F. tularensis* subsp. *mediasiatica* registered only in the Shu-Ili basin, where *F. tularensis* subsp. *holarctica* also circulates. Despite the extensive distribution of foci containing *F. tularensis* subsp. *mediasiatica*, no cases of human infection have been reported.

In agreement with previous reports regarding *F. tularensis*, the evolution of subspecies *mediasiatica* appears to be clonal. Varying levels of homoplasy are observed among bacterial species. For instance, in a global sample of 36 *C. trachomatis* genomes, 17,163 SNPs were identified, of which 4492 (26%) were homoplastic and caused by interstrain recombinations [37]. The homoplasia levels observed are reminiscent of observations in other highly monomorphic and clonal species such as *Bacillus anthracis* [38]. The present study highlights the remarkably low genetic diversity among 53 genomes of *F. tularensis* subsp. *mediasiatica* isolated over a significant time span and across territories separated by thousands of kilometers, which might indicate that these territories constitute a single ecosystem for the subspecies. A total of 330 SNPs were identified among the 53 available genomes of *Francisella tularensis* subsp. *mediasiatica*. The genetic diversity of other *Francisella* subspecies significantly surpasses that found in *mediasiatica*. An analysis of the core genome among *F. tularensis* subsp. *holarctica* strains isolated in Germany revealed that the average SNP distance between two isolates was 226.6, ranging from 0 to 490 SNPs [39]. Globally, the genetic diversity within the three main *holarctica* sublineages (B.4, B.6, and B.12) exceeds 2000 SNPs [16].

MLVA genotyping demonstrated a high discriminatory ability despite the genetic homogeneity of the studied strains according to wgSNP analysis. Simpson’s discrimination index among the 28 studied strains was calculated to be 0.9497, based on a panel of eleven loci. Discrimination index values falling within the range of 0.95–0.99 indicate that the methods used are considered “ideal” for genotyping [40,41]. In our sample of *F. tularensis* subsp. *mediasiatica*, the most significant discriminatory power was observed in the VNTR loci Ft-M03, Ft-M22, and Ft-M05. Curiously, the last two markers exhibit low or zero variability among the subspecies *F. tularensis* and *F. holarctica*, [16,42,43]. Out of the 18 identified MLVA11 genotypes, only five contained two or more strains, with these genotypes clustering strains isolated from a single region during a specific period of time. This suggests the suitability of the MLVA typing method as the primary tool for discriminating *F. tularensis* subsp. *mediasiatica* in epidemiological control. However, the topology of the MLVA and wgSNP trees is not directly comparable. While for most strains, the clustering obtained with MLVA aligns with wgSNP, there are exceptions, notably strains Ftm-S9 and Ftm-S10. These strains exhibit the same genotype according to wgSNP but differ at three VNTR loci. It is important to note that wgSNP reliably reflects the phylogenetic and evolutionary relationships, as evidenced by consistent clustering based on a single focus and time of isolation. Therefore, it is essential to interpret with caution the genetic diversity of strains identified by MLVA, especially when isolated from the same outbreak. MLVA is not a robust phylogenetic tool.

Based on wgSNP analysis, 53 strains of the *F. mediasiatica* subspecies clustered into three previously proposed lineages [22,34]. Within these, the 28 strains from our study were assigned to the M.I lineage, alongside seven previously sequenced strains conserved in Russia and Sweden [22,44,45]. Sublineages M.II and M.III consist of strains isolated in Russia and Uzbekistan, respectively. Considering the historical recognition of two regions with the circulation of the *F. mediasiatica* subspecies—the Ili and Shu river basins in Kazakhstan, and the Amu Darya river basin in Uzbekistan—the clustering of strains from Kazakhstan and Uzbekistan into different sublineages M.I and M.III allows us to tentatively assign strains without precise geolocation data (FSC148, FSC149, and FSC122 from the Swedish collection) to the Kazakhstan focus.

The analysis using wgSNP failed to establish clear associations with active tularemia foci and the isolation period. For instance, the most numerous sublineage, M.I_4, comprised 21 strains isolated from at least five outbreaks spanning a distance of 400 km and isolated over forty years from 1965 to 2004. Previously, Timofeev and co-authors reported the isolation of nearly identical strains of the *mediasiatica* subspecies from two independent foci separated by a distance of 500 km [21]. This finding suggests the potential involvement of birds in the spread of *F. tularensis*, as birds can act as carriers of ticks [46,47] or of the pathogens themselves [48,49]. Our study highlights the circulation of genetically similar strains not only across distant foci but also over extended periods, underscoring the persistent nature of these strains. The present investigation illustrates how the Shu and Ili rivers appear to constitute a single ecosystem within which M.I strains circulate. This circulation would allow us to explain the limited genetic diversity observed, resulting from random genetic drift and periodic selection events.

## Figures and Tables

**Table 1:** Simpson’s Diversity Index for 11 VNTR loci.

Locus	Allele Number	Total *n* = 27	Standard Deviation
Ft-M03	14	0.9153	[0.8619, 0.9688]
Ft-M22	7	0.8016	[0.6970, 0.9062]
Ft-M05	7	0.7963	[0.7129, 0.8797]
Ft-M06	2	0.1376	[0.0000, 0.3044]
Ft-M20A	2	0.0714	[0.0000, 0.2017]
Ft-M02	1	0.0000	[0.0000, 0.0000]
Ft-M04	1	0.0000	[0.0000, 0.0000]
Ft-M10	1	0.0000	[0.0000, 0.0000]
Ft-M20B	1	0.0000	[0.0000, 0.0000]
Ft-M23	1	0.0000	[0.0000, 0.0000]
Ft-M24	1	0.0000	[0.0000, 0.0000]
MLVA-11	18	0.9497	[0.9069, 0.9926]

## Data Availability

All data from this project are publicly available. NCBI Bioproject Accession: PRJNA639508; Sequence Read Archive (SRA) Accessions: SRR29157962-SRR29157988, SRR29374495.

## Supplementary Materials

The following supporting information can be downloaded at: https://www.mdpi.com/article/10.3390/pathogens13070581/s1, Figure S1: *F. tularensis mediasiatica* genetic diversity reflected by wgSNP analysis; Table S1: Metadata 27 strains *Francisella tularensis* subsp. *mediasiatica* from Kazakhstan; Table S2: Metadata worldwide strains *Francisella tularensis* subsp. *mediasiatica*.
