# Supplementary material for: Genetic Homogeneity of Francisella tularensis subsp. mediasiatica Strains in Kazakhstan
Source: Pathogens. 2024 Jul 12;13(7):581. doi: 10.3390/pathogens13070581 (PMC11279412; doi:10.3390/pathogens13070581)
Supplement: Supplementary file 1 [file pathogens-13-00581-s001.zip › Figure S1.pdf]

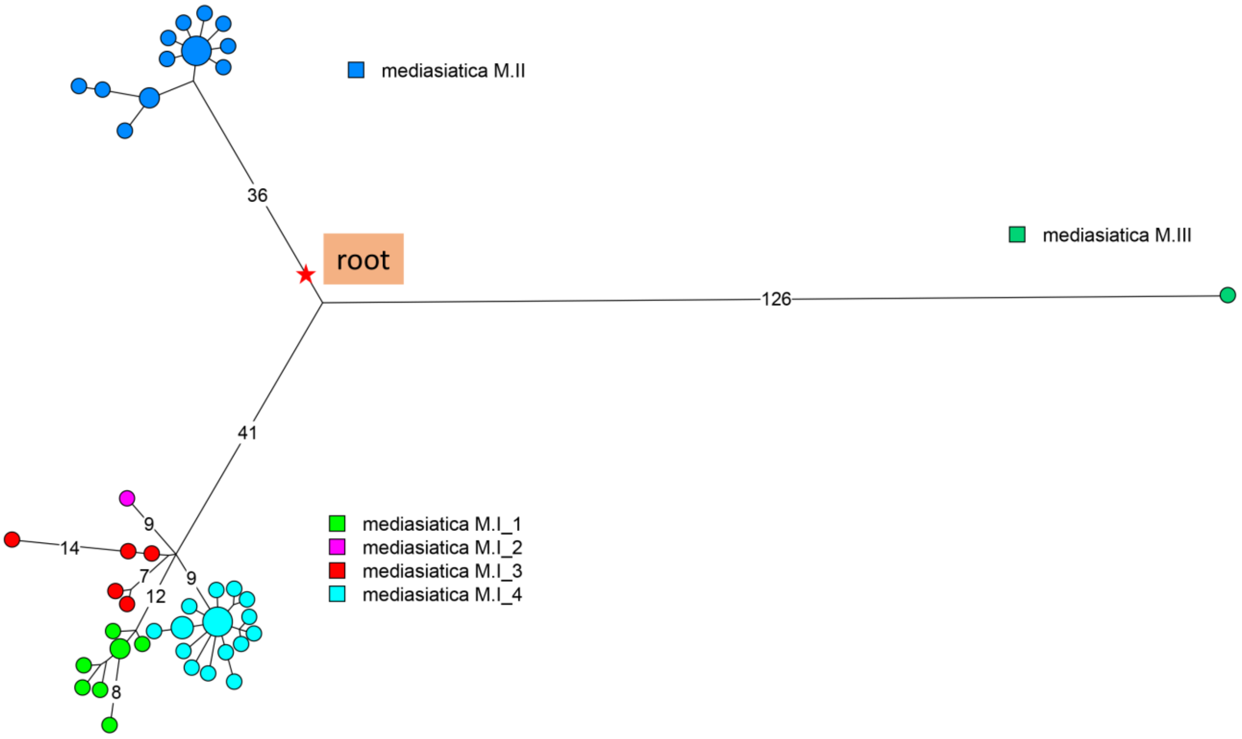

Figure S1. *F. tularensis mediasiatica* genetic diversity reflected by wgSNP analysis. Fifty-three strains were included (28 from this project and 25 from public repositories). The maximum parsimony tree was based upon 330 core-genome SNPs, tree size is 330 (no homoplasia). Branch lengths above five are indicated. Nodes are colored according to clade (M.II, M.III) or subclade (within M.I).
